# Esculetin and its derivatives: recent advances in efficacy, mechanisms, and translational challenges

**DOI:** 10.1007/s00210-026-05149-4

**Published:** 2026-03-26

**Authors:** Noha M. Gamil, Rawan Atef Essmat, Fatma Shaban Hafez, Osama A. Alaziz, Asmaa E. Bogor, Shehab Wageh, Donia G. Youssef, Nourhan Abdellatif, Riham A. El-Shiekh, Samar S. Khalaf

**Affiliations:** 1 Department of Pharmacology and Toxicology, College of Pharmaceutical Sciences and Drug Manufacturing, Misr University for Science and Technology (MUST), P.O. Box 77, Giza, Egypt; 2 Faculty of Pharmacy, Modern University for Information and Technology, Cairo, 11728 Egypt; 3 Chemistry and Zoology Department, Faculty of Science, Menoufia University, Ashmoun, 32839 Egypt; 4 Biotechnology Department, Faculty of Science, Mansoura University, Mansoura, 35516 Egypt; 5 Biochemistry Department, Faculty of Science, Zagazig University, Zagazig, Egypt; 6 Botany and Chemistry Department, Faculty of Science, Ain Shams University, Cairo, Egypt; 7 Physics Department, Faculty of Women for Arts, Science and Education, Ain Shams University, Cairo, Egypt; 8 School of Biotechnology, Badr University in Cairo (BUC), Badr City, Cairo, 11829 Egypt; 9 Department of Biochemistry, Faculty of Pharmacy, Heliopolis University, Cairo, 11785 Egypt; 10 Department of Pharmacognosy, Faculty of Pharmacy, Cairo University, Cairo, 11562 Egypt

**Keywords:** Anticancer activities, Antioxidant, Anti-inflammatory, Coumarin derivatives, Clinical translation, Esculetin, Nutraceuticals

## Abstract

Esculetin (ESC, 6,7-dihydroxycoumarin), a naturally occurring polyphenolic coumarin found in numerous medicinal and dietary plants, has emerged as a multi-target bioactive scaffold demonstrating anti-inflammatory, antioxidant, and antiproliferative properties in diverse preclinical models. Recent investigations reveal that ESC modulates critical signaling pathways, including NF-κB, Nrf2/antioxidant response element (ARE), and mitochondrial apoptotic cascades, across multiple disease contexts encompassing dermatology, metabolic disorders, cardiovascular disease, neurodegeneration, hepatorenal protection, and malignant transformation. Structural modification of the coumarin ring system yields derivatives with enhanced target selectivity and improved metabolic stability in select compounds. However, ESC is substantially limited by poor aqueous solubility, rapid phase II metabolism, and an estimated low oral bioavailability (5–15%), representing a critical barrier to therapeutic translation. Notably, no phase II/III clinical trials have been completed, and human safety and efficacy data remain virtually absent. Recent innovations, including cocrystal technology, nanoparticle formulations, and rational structural optimization, demonstrate promise in preclinical contexts. This comprehensive review synthesizes pharmacological evidence across disease indications, evaluates bioavailability enhancement strategies, explicitly discusses evidence limitations, addresses safety considerations, and outlines translational research priorities essential for clinical or nutraceutical development.

## Introduction

Nutraceuticals have gained prominence as preventive and therapeutic agents for chronic disease management, offering complementary approaches to conventional pharmacotherapy (Garg et al. 2022). Among these, esculetin (ESC), a naturally occurring coumarin derivative also known as 6,7-dihydroxycoumarin, distinguishes itself by a comprehensive spectrum of biological activities. ESC has been extensively characterized for its anti-inflammatory, antioxidant, anticoagulant, and anticancer properties, with emerging mechanistic evidence supporting diverse clinical applications (Shen et al. 2023; Zhang et al. 2022a, b).

Recent decades have witnessed substantial progress in elucidating ESC’s molecular mechanism of action. Preclinical research integrating both in vitro and in vivo methodologies has delineated how ESC and its chemically modified derivatives influence critical cellular signaling cascades. Notably, ESC’s suppression of the NF-κB (nuclear factor kappa-B) pathway has been causally linked to attenuated inflammatory responses and reduced oxidative stress (OS), thereby modulating apoptotic cell death (Li et al. 2022; Zhang et al. 2022a, b). Furthermore, structure–activity relationship (SAR) investigations of ESC derivatives have demonstrated that rational structural modifications enhance therapeutic efficacy and bioavailability, parameters essential for translating discoveries into clinical applications (Garg et al. 2022).

Contemporary synthetic approaches have yielded novel ESC variants with improved pharmacokinetic and safety profiles, thereby expanding therapeutic utility in nutraceutical and pharmaceutical contexts (Garg et al. 2022). This research establishes ESC’s multifaceted roles in disease pathophysiology and supports its integration into comprehensive health management strategies.

### Botanical sources and phytochemical distribution

Esculetin is widely distributed across medicinal and culinary plant species and exhibits pharmacological effects, including anti-inflammatory, antioxidant, anticoagulant, and anticancer activities. Phytochemical databases, including Dr. Duke’s Phytochemical and Ethnobotanical Database and IMPPAT (Indian Medicinal Plants, Phytochemistry, and Therapeutics), document ESC occurrence in numerous plant genera (Duke 2017; Sinha et al. 2022; Vivek-Ananth et al. 2023).

Key botanical sources include *Aesculus hippocastanum* (horse chestnut), traditionally used for vascular insufficiency; *Cichorium intybus* (chicory), recognized for its hepatoprotective properties; *Artemisia capillaris*, used in traditional Asian medicine for hepatoprotective and antimicrobial effects; *Anethum graveolens* (dill), a culinary herb that provides dietary coumarins; and *Citrus paradisi* (grapefruit), which contains high levels of flavonoids and coumarins (Duke 2017). Recognition of these botanical sources, supported by comprehensive phytochemical databases, underscores ESC’s widespread natural occurrence and emphasizes its importance in medicinal plant research and pharmacognosy. Knowledge of ESC’s natural distribution across diverse plant species provides valuable perspectives for developing phytopharmaceutical formulations and dietary supplements (Table 1).

**Table 1:** Medicinal plants and their utilized parts for the extraction of esculetin-containing bioactive compounds, with their typical use (dietary/medicinal) and qualitative relevance

Plant (Latin name)	Common/traditional name	Typical use (dietary/medicinal)	Main plant parts used	Qualitative relevance for ESC (H/M/L)
*Aesculus hippocastanum*	Horse chestnut	Vascular/venous insufficiency preparations	Bark, seeds	High
*Cichorium intybus*	Chicory	Hepatoprotective, digestive tonic, functional food	Aerial parts, roots, leaves	High
*Artemisia capillaris*	–	Traditional hepatoprotective and antibacterial remedies	Whole plant	High
*Anethum graveolens*	Dill	Culinary herb, digestive aid	Fruits, seeds	Moderate
*Atropa belladonna*	Deadly nightshade	Historical antispasmodic/mydriatic (toxic)	Leaves, whole plant	Moderate
*Citrus paradisi*	Grapefruit	Dietary fruit, cardiometabolic benefits	Fruits	Moderate
*Citrus reticulata*	Mandarin orange	Dietary fruit, digestive/respiratory uses	Fruits	Moderate
*Fraxinus* spp. (*F. ornus*, *F. rhynchophylla*, *F. floribunda*)	Manna ash, ash species	Traditional laxative, hepatobiliary and tonic uses	Bark, leaves	High
*Taraxacum campylodes*	Dandelion	Hepatobiliary and digestive uses	Aerial parts, roots	Moderate
*Theobroma cacao*	Cacao	Cocoa/chocolate, functional food	Seeds	Low–Moderate
*Hordeum vulgare*	Barley	Cereal grain, functional food	Whole plant	Low–Moderate

H = High: well-established ESC source with notable traditional/modern use and clearer pharmacological relevance; M = Moderate: documented ESC source with relevant but less central role; L = Low: documented ESC presence but limited direct ESC-focused evidence or secondary in typical use

### Literature search methodology

This review was conducted through systematic searches of PubMed/MEDLINE, Scopus, and Web of Science, complemented by searches of specialized phytochemical databases (Dr. Duke’s Phytochemical and Ethnobotanical Database, IMPPAT). Primary search terminology included “esculetin,” “6,7-dihydroxycoumarin,” and “cichorigenin,” combined with disease-specific or mechanism-based terms including “esculetin AND inflammation,” “esculetin AND cancer,” “esculetin AND diabetes,” “esculetin AND antioxidant,” “coumarin AND bioavailability,” and “coumarin AND derivatives.” Disease-specific searches incorporated relevant MeSH headings (neoplasms, cardiovascular diseases, diabetes mellitus, neurodegeneration, and hepatic/renal disorders). The search focused on publications from 2010 to 2025, with particular attention to recent literature (2018–2025). Seminal studies published before 2010 were retained, as they were foundational to understanding ESC biology.

The inclusion criteria included original experimental studies (e.g., in vitro cell culture, animal models, and human studies); review articles and systematic reviews for contextual understanding; publications in English; and studies that directly measure esculetin or its derivatives with pharmacological, mechanistic, chemical, or translational relevance. However, studies on other coumarins that do not specifically address ESC, opinion pieces or editorials lacking original data, and redundant publications, reports, or presentations are also excluded.

## Chemical composition, synthesis, and structure-elucidation

### Molecular composition

Esculetin (6,7-dihydroxycoumarin) is a naturally occurring hydroxycoumarin with an aromatic lactone structure. The hydroxylated benzopyranone core has been confirmed through multimodal spectroscopic techniques, including nuclear magnetic resonance (NMR), mass spectrometry (MS), infrared spectroscopy (IR), and ultraviolet–visible spectroscopy (UV–Vis). Key physicochemical parameters are as follows: molecular formula: C₉H₆O₄; molecular weight: 178.14 g/mol; melting point: 270 °C; aqueous solubility: ~ 10 mg/mL (severely limited); hydrogen bond donors: 2 (hydroxyl groups at positions 6 and 7); hydrogen bond acceptors: 4 (lactone and hydroxyl oxygen); rotatable bonds: 0 (planar configuration) (Garg et al. 2022).

### Chemical synthesis

Esculetin has been synthesized through multiple chemical routes. Yang and colleagues reported microwave-assisted synthesis utilizing zinc chloride (ZnCl₂) as a catalyst. A combination of 1,2,4-benzenetriol, ethyl propionate, and ZnCl₂ (1:1:3.5 g ratio) subjected to microwave irradiation at 105 °C for 10 min (400 W) yielded approximately 87.4% ESC (Yang and Gao 2011). Alternative synthetic approaches have been described by Zhang and associates, wherein p-benzoquinone was reacted with concentrated sulfuric acid and acetoacetate (0.15:3:1 molar ratio) at 45 °C for 3 h, producing peracetylated 1,2,4-benzenetriol. Subsequent catalysis with concentrated sulfuric acid and malic acid yielded ESC with an overall yield of 80% (Cao et al. 2013). Additional biosynthetic pathways have been elucidated, demonstrating glucose conversion through multiple intermediates, including pyruvate, phosphoenolpyruvate, erythrose-4-phosphate, shikimate-3-phosphate, and chorismate, proceeding through enzymatic steps catalyzed by prephenate dehydrogenase, prephenate dehydratase, and related enzymes to yield the final coumarin product (Yang et al. 2015).

### Structure–activity relationships and derivative optimization

Rational structural modification of the ESC scaffold has been pursued to enhance pharmacokinetic properties and therapeutic efficacy. ESC exhibits dose-dependent growth inhibition of human melanoma G361 cells by suppressing the Sp1 transcription factor, thereby affecting downstream targets (p27, p21, cyclin D1) integral to cell cycle regulation. ESC activates apoptotic signaling by enhancing caspase-3 and PARP cleavage (Anand et al. 2013).

Therapeutic limitations imposed by rapid phase II metabolism have motivated the development of C-4- and C-8-substituted compounds via the von Pechmann and Mannich reactions. In vitro testing on A549 and B16 cell lines identified 8-(pyrrolidin-1-ylmethyl)−4-trifluoromethyl ESC as the optimal derivative, demonstrating 20-fold enhanced antiproliferative activity and threefold prolonged half-life in human liver S9, with structural analysis revealing that nitrogen-containing groups at C-8 substantially enhance both metabolic stability and anticancer potency (Pan et al. 2015; Zhang et al. 2022a, b).

A recent investigation of C-4-, C-8-, and position-7-substituted derivatives in HepG2.2.15 hepatitis B-expressing cells, using ELISA and MTT assays (with lamivudine as the control), demonstrated moderate anti-HBV activity. Morpholine groups significantly reduced HBeAg expression, while 2-methylimidazole derivatives suppressed HBsAg levels. Compound 4a exhibited maximal anti-HBeAg activity (IC₅₀ = 15.8 ± 4.2 µM), whereas compound 6 d was most effective for HBsAg inhibition (IC₅₀ = 21.4 ± 2.8 µM), with several molecules demonstrating improved metabolic stability relative to parent ESC (Ye et al. 2023).

## Common molecular mechanisms of esculetin

### NF-κB pathway modulation

The NF-κB signaling cascade constitutes a central regulatory axis controlling pro-inflammatory gene expression. In unstimulated cells, NF-κB (composed of p65 and p50 subunits) remains sequestered in the cytoplasm through association with inhibitory IκBα proteins. Upon inflammatory stimulation, IκBα undergoes phosphorylation, ubiquitination, and proteasomal degradation, thereby liberating NF-κB for nuclear translocation and binding to κB elements in the promoters of target genes (TNF-α, IL-6, IL-1β, iNOS, COX-2) (Sun et al. 2019).

ESC suppresses this pathway via several mechanisms. It stabilizes IκBα, preventing its phosphorylation and degradation; reduces p65 phosphorylation; and prevents p65 from entering the nucleus. These actions lead to lower levels of pro-inflammatory mediators, such as TNF-α, IL-6, nitric oxide, and prostaglandins, thereby interrupting the escalation of the inflammatory response (Garg et al. 2022).

### Nrf2/antioxidant response element pathway

The Nrf2/ARE pathway constitutes the primary cellular defense mechanism against oxidative stress. Under basal conditions, Nrf2 associates with Keap1 (Kelch-like ECH-associated protein 1) in the cytoplasm. Upon oxidative or electrophilic stress, Nrf2 dissociates from Keap1, accumulates, translocates to the nucleus, and heterodimerizes with small musculoaponeurotic fibrosarcoma (sMAF) proteins. This heterodimer binds ARE sequences in target gene promoters, inducing transcription of antioxidant and cytoprotective genes, including NAD(P)H quinone oxidoreductase 1 (NQO1), heme oxygenase-1 (HO-1), glutathione S-transferases (GSTs), and γ-glutamylcysteine synthetase (γ-GCS) (Jayakumar et al. 2022).

ESC activates this pathway, leading to higher expression of Nrf2 target genes and increased activity of antioxidant enzymes. This dual approach, direct ROS scavenging through electron donation and indirect antioxidant enhancement via Nrf2 activation, explains ESC’s effectiveness in various cases of oxidative stress-related diseases (Jayakumar et al. 2022).

### Mitochondrial apoptotic cascade

Mitochondria execute intrinsic apoptosis through pro-apoptotic BAX and BAK activation at the outer mitochondrial membrane, culminating in mitochondrial outer membrane permeabilization (MOMP). This permits cytochrome c release into the cytosol, where it binds to apoptotic protease-activating factor 1 (APAF-1), forming the apoptosome complex that recruits and activates initiator caspase-9. Active caspase-9 cleaves and activates effector caspases (caspase-3, caspase-7), which proteolytically process poly (ADP-ribose) polymerase (PARP), leading to DNA fragmentation, chromatin condensation, and programmed cell death (Jiang et al. 2021a, b).

ESC promotes apoptosis through enhanced mitochondrial ROS generation (particularly in cancer contexts), increased BAX/BAK expression or activity, reduced anti-apoptotic protein expression (Bcl-2, Bcl-xL), and direct caspase cascade activation (Garg et al. 2022).

### Reactive oxygen species metabolism

Reactive oxygen species (ROS), including superoxide (O₂•⁻), hydrogen peroxide (H₂O₂), and hydroxyl radical (•OH), are generated during normal mitochondrial respiration and are further amplified in pathological contexts (e.g., inflammation, cancer, metabolic dysfunction). Excessive ROS damages cellular macromolecules (DNA, proteins, lipids), compromising cell viability and organ function (NaHee et al. 2018).

ESC functions as an antioxidant through dual mechanisms: direct ROS scavenging via hydroxyl groups at positions 6 and 7 of the coumarin ring, which donate electrons to ROS, converting them to inert products, and indirect antioxidant upregulation via Nrf2/ARE pathway activation. ESC increases expression and activity of antioxidant enzymes (superoxide dismutase [SOD], catalase [CAT], glutathione peroxidase [GPx]), which catalytically neutralize ROS (Garg et al. 2022; Jayakumar et al. 2022).

## Pharmacological activities across disease contexts (Table 2)

**Table 2:** Summary of esculetin pharmacological activities across disease models

Disease/condition	Model(s)	Mechanism(s)	Efficacy* level	Evidence gaps	Citations
Cancer (multiple types: liver, colorectal, others)	Liver cancer (H22, HepG2, mouse xenografts); colorectal cancer (HCT116, HT-29, xenografts); various cell lines	Inhibits PI3K/Akt/mTOR and ENO1; targets GPI and glycolytic enzymes; induces mitochondrial apoptosis (↑ Bax, caspase-3/9; ↓ Bcl-2); anti-angiogenic (↓ VEGF); multi-pathway modulation (Wnt/β-catenin, MAPK, JAK/STAT3); ferritinophagy; lipid peroxidation; copper homeostasis; epithelial-mesenchymal transition	Moderate–high preclinical efficacy (robust in vitro and several in vivo mouse data)	No human trials; long-term safety, optimal dosing, and resistance mechanisms are unclear	(Hong et al. 2025; Jiang and Bao 2025; Li et al. 2022; Ma et al. 2025)
Epilepsy/seizures	PTZ- and penicillin-induced seizures in rats	Anti-neuroinflammatory (↓ IL-1β, IL-6, NF-κB, activin-A); improves redox balance and survival signaling	Moderate (single well-designed rodent study)	Lack of chronic epilepsy models, pharmacokinetics in the brain, and clinical data	(Danis et al. 2023; Zhang et al. 2022a, b)
Schizophrenia	Ketamine-induced schizophrenia in mice	Attenuates oxidative stress and neuroinflammation; normalizes dopamine, serotonin, glutamate, ↑ BDNF; modulates NMDA-related pathways	Moderate (behavioral + biochemical changes in one model)	No other psychosis models; target specificity and human relevance are uncertain	(Khalid et al. 2024)
Neurodegeneration (AD-like)	SH-SY5Y neurons exposed to Aβ oligomers (Aβ1–42, Aβ25–35); D-galactose AD rats; AlCl₃ cognitive-deficit rats	Acts as a bifunctional antioxidant: direct ROS scavenging plus Nrf2 activation and ↑ GSH; activates ERK/Akt-Nrf2 signaling; protects against Aβ-induced oxidative stress, mitochondrial dysfunction, and apoptosis; improves cognitive performance, antioxidant enzymes, cholinergic function, and reduces AGEs and inflammatory markers in AD-like rats 1,261,012	Emerging–moderate (multiple in vitro Aβ studies; several toxin/aging AD-like rat models)	No transgenic AD models; no data on β-amyloid or tau burden in vivo; pharmacokinetics in brain and long-term safety unclear; no human studies	(Ahmed et al. 2023; Gao and Gong 2018; Journal 2023; Pruccoli et al. 2020)
Neurodegeneration (PD/HD spectrum)	MPTP PD mice; 3-nitropropionic acid HD-like rats	Reduces MPTP-induced dopaminergic neuron loss, nitrosative stress (3-nitrotyrosine), caspase-3 activation, and maintains GSH in substantia nigra; protects against 3-NP-induced motor and cognitive deficits and striatal oxidative stress by restoring SOD, CAT, GSH, GPx and lowering lipid peroxidation and protein carbonyls 814	Emerging (few but consistent rodent toxin models)	No 6-OHDA PD models reported for esculetin; mechanisms beyond generic antioxidant effects (e.g., α-synuclein aggregation, ferroptosis) poorly defined; no human data	(Karandikar and Thangarajan 2017; Subramaniam and Ellis 2013)
Lupus nephritis	MRL/lpr autoimmune mice	Inhibits complement activation (blocks C3 convertase C4b2a); activates Nrf2 anti-oxidant genes; suppresses NF-κB and TGF-β/Smad3 profibrotic signaling	Moderate (functional, histologic kidney protection in one chronic model)	Translation to human LN, combination with standard immunosuppression untested	(Zhang et al. 2022a, b)
Osteoarthritis	MIA-induced OA in rats; LPS-stimulated RAW264.7 cells	↓ NO, PGE₂, TNF-α, IL-1β, IL-6; protects cartilage, improves weight-bearing; NF-κB/MAPK suppression inferred	Moderate (single in vivo model plus in vitro support)	No large-animal or clinical OA data; disease-modifying potential vs. symptomatic relief unknown	(Chen et al. 2022; Garg et al. 2022; Ju et al. 2024)
Atherosclerosis/cardiovascular	Reviews, various in vivo and in vitro vascular models	Antioxidant; ↓ triglycerides; inhibits VSMC proliferation, MMP-9, LDL oxidation; improves HDL-C efflux; anti-platelet via PLCβ2–PKC–Akt inhibition	Moderate (multiple preclinical models; good mechanistic support)	Few integrated atherosclerosis models; no outcome studies or clinical data	(Hsia et al. 2019; Wang et al. 2022; Zhang et al. 2022a, b)
Cardiac protection (acute MI/ischemia–reperfusion)	Isoproterenol (ISO) MI in rats; myocardial I/R in rats; H9c2 cardiomyoblasts	Restores SOD, CAT, GSH; ↓ MDA and lipid peroxidation; ↓ CK‑MB, LDH; stabilizes lysosomal and Na⁺/K⁺‑ATPase activity; ↓ TNF‑α, IL‑6, NF‑κB expression; ↓ apoptosis (↓ Bax, caspase‑3/9; ↑ Bcl‑2); inhibits RIP140/NF‑κB inflammatory pathway	Limited–moderate (two main in vivo esculetin MI/I‑R studies with supportive in vitro data)	No dose‑finding or chronic post‑MI remodeling data; no combination studies with standard MI therapies (reperfusion, β‑blockers, ACEi/ARB/ARNI, SGLT2i); no pharmacokinetics in injured myocardium; no human data	(P et al. 2021; Pullaiah et al. 2021; Weiwei et al. 2016; Zhang et al. 2024)
Autoimmune/inflammatory bowel disease, colitis	TNBS and DSS-induced colitis in rats and mice; HCT116 and other cells	Inhibits NF-κB and MAPKs; ↓ TNF-α, IL-6, IL-1β, MPO, COX-2, iNOS; activates Nrf2/HO-1 and HIF-1α in some models	Moderate (multiple gut-inflammation models)	Long-term relapse models, microbiome effects, and clinical IBD trials are lacking	(Cai and Cai 2023; Garg et al. 2022; Ju et al. 2024)
Intervertebral disc degeneration	IL-1β–treated human nucleus pulposus cells	Activates Nrf2/HO-1; inhibits NF-κB; ↓ ROS, MDA, iNOS, IL-6, TNF-α, MMP-3/13; preserves collagen II and aggrecan	Low–moderate (only cell-based data)	No animal IVDD models or in vivo pharmacokinetics in disc tissue	(Huang et al. 2024)
Intestinal ischemia/reperfusion injury	Rat intestinal I/R model; epithelial H/R cells	Binds SIRT3 and activates SIRT3/AMPK/mTOR; ↓ inflammation, oxidative stress, apoptosis; ↑ autophagy	Moderate (coherent in vivo and in vitro data)	Dose–response, timing of administration, and comparison with standard I/R therapies are unclear	(Shen et al. 2023)
Intracerebral hemorrhage/ferroptosis	Hemin-induced PC12 cells; ICH mouse model	Inhibits ferroptosis via ↑ NUDT1-mediated m7G methylation and GPX4 stability; ↓ Fe-dependent lipid ROS, MDA	Low–moderate (new, limited models)	Early preclinical stage; broader stroke models and human relevance not yet known	(Gong et al. 2025)
Systemic inflammatory, oxidative stress–related diseases (overview)	Numerous cell and animal models (review)	Broad antioxidant via Nrf2; anti-inflammatory via NF-κB and MAPK inhibition; immunomodulatory, antidiabetic, antibacterial	Evidence is strong at the preclinical level across indications	Lack of standardized dosing, bioavailability solutions, and rigorous clinical trials across diseases	(Cai and Cai 2023; Ju et al. 2024; Zhang et al. 2022a, b)
Skin inflammation	Imiquimod-induced psoriasis-like dermatitis, HDM/DNCB-induced atopic dermatitis in mice	Inhibits NF-κB and STAT1/MAPK; reduces TNF-α, IFN-γ, IL-4, IL-6, IL-13, IL-17, IL-22, IL-23, IL-31; induces CD4⁺Foxp3⁺ Tregs	Supported (multiple rodent studies)	No human clinical trials; optimal dose, long-term safety, and topical vs systemic delivery unknown	(Cai and Cai 2023; Chen et al. 2018; Ju et al. 2024)
Wound healing	No direct esculetin wound-closure models found; related coumarin esculin improves full-thickness murine skin wounds	Esculin: activates Wnt/β-catenin, increases collagen I/III, PCNA, CD31, granulation tissue, epithelial thickness	Indirect/limited (structural analog, not esculetin)	Direct in vivo wound-healing studies with esculetin lacking; unclear if esculetin shares esculin’s pro-healing profile	(Ju et al. 2024; Li et al. 2022; Xu et al. 2025)
Type 2 diabetes/metabolic dysfunction	STZ-induced diabetic rats; STZ + high-fat diet diabetic mice; in vitro adipocytes, hepatocytes	Lowers blood glucose and HbA1c; increases insulin; decreases hepatic G6Pase and G6Pase/GLK ratio; improves antioxidant enzymes; reduces inflammatory cytokines; modulates AMPK, Nrf2 and lipid synthesis genes	Supported in rodents and cell models	No human trials; pharmacokinetics show low oral bioavailability; clinically relevant dosing and long-term metabolic safety undetermined	(Cai and Cai 2023; Choi et al. 2016; Garg et al. 2022; Prabakaran and Ashokkumar 2013; Zhang et al. 2022a, b)
Hepatoprotection (NAFLD, toxic/hepatic injury)	Diabetic mice with HFD-induced NAFLD; primary rat hepatocytes with FFA-induced lipotoxicity; DOX-induced hepatotoxicity rats; H₂O₂-injured HepG2 cells	Antioxidant via Nrf2/ARE, ↑SOD, GPx, GSH; ↓ROS and MDA; inhibits JNK; activates AMPK to reduce SREBP1c and lipogenesis; downregulates Fasn, Dgat2, Plpp2 and inflammatory genes (Tlr4, Myd88, Nfkb, Tnfα, Il6); reduces lipid accumulation and necrosis	Strong preclinical support (multiple in vivo and in vitro studies)	No human hepatoprotection studies; interaction with standard NAFLD/NASH therapies, and efficacy in viral or autoimmune liver disease not defined	(Cai and Cai 2023; Choi et al. 2016; Garg et al. 2022; Köroğlu et al. 2024; Luo et al. 2024; Xia et al. 2023)

*Efficacy level is based on quantity and quality of preclinical evidence, not clinical data

### Dermatological applications

**Fig. 1:**
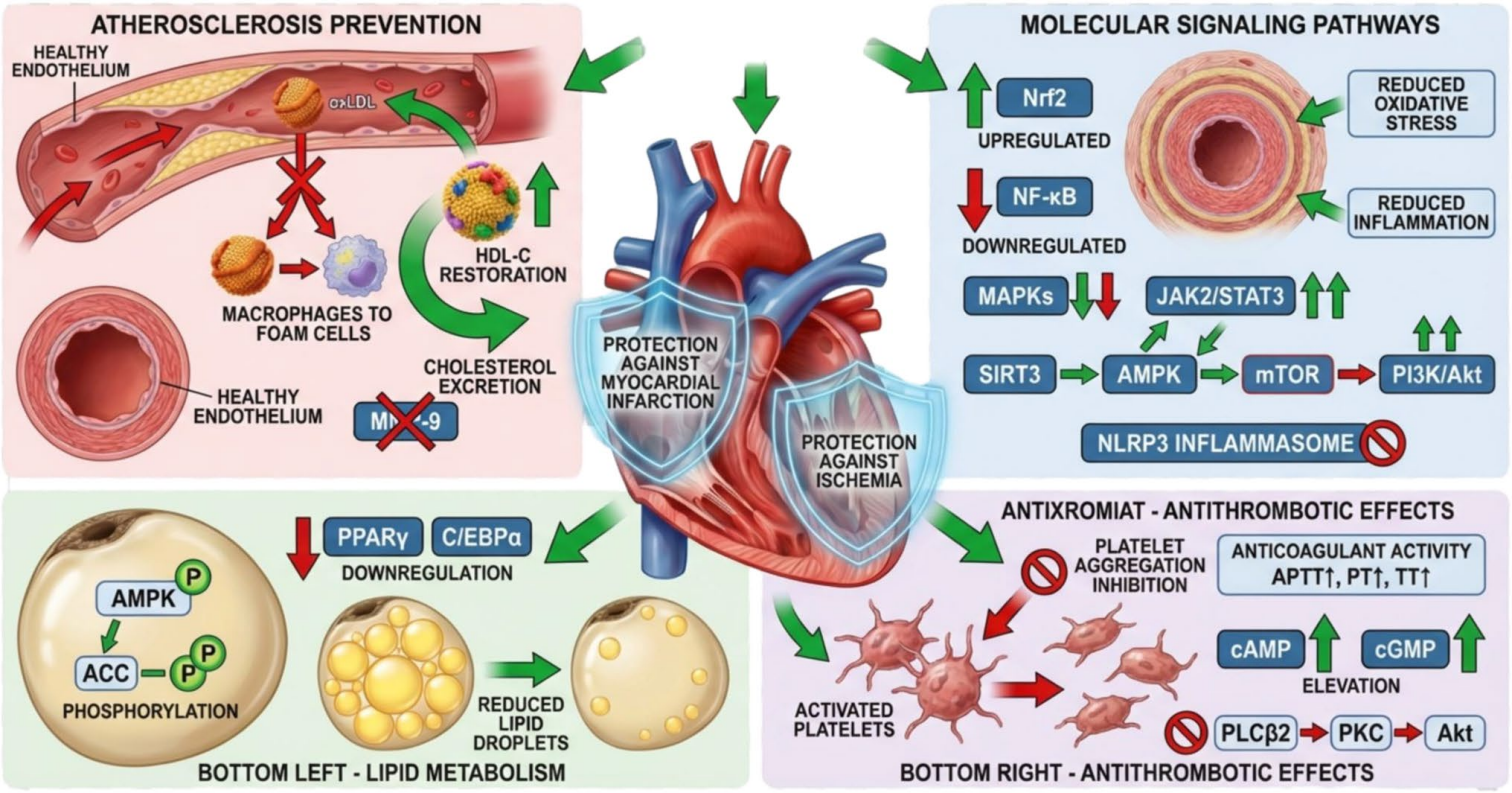
Multifaceted protective and reparative effects of esculetin (ESC) on skin physiology and pathology.This schematic illustrates the principal mechanisms by which ESC improves skin health across three domains:** A** photoprotection via ROS scavenging and lipid peroxidation inhibition (Hong et al. 2014), preventing UV-induced DNA damage and collagen/elastin degradation; **B** wound healing enhancement through elevated VEGF and TGF-β1 expression, promoting keratinocyte migration, fibroblast proliferation, and extracellular matrix remodeling (Garg et al. 2022); **C** anti-inflammatory effects in eczema, psoriasis, and dermatitis via NF-κB pathway inhibition and pro-inflammatory cytokine (IL-6, TNF-α) reduction (Zhen et al. 2019). All arrows represent validated experimental findings in cell culture and animal models; no speculative connections are shown. Data derive primarily from in vitro keratinocyte/fibroblast models and topical application in murine inflammation models; human clinical efficacy remains to be established

Skin disorders, including chronic inflammation (eczema, psoriasis, dermatitis), photodamage from ultraviolet exposure, and impaired wound healing, represent significant healthcare burdens affecting quality of life (Diepgen et al. 2012; Landén et al. 2016). ESC addresses three major categories of skin concern (photoprotection, wound healing, and inflammatory control) through complementary molecular mechanisms. These mechanisms are schematically illustrated in Fig. 1 and discussed in detail below.

#### Anti-inflammatory effects

ESC demonstrates pronounced anti-inflammatory activity in epidermal cells. Studies in basal-layer keratinocytes exposed to pro-inflammatory stimuli (TNF-α, lipopolysaccharide) document ESC-mediated decreases in inflammatory markers (IL-6, IL-8, TNF-α, iNOS, COX-2) (Çınar et al. 2025; Zhen et al. 2019). In animal models of inflammatory dermatitis, topical ESC application effectively reduces erythema, edema, and inflammatory cell infiltration, supporting potential clinical utility in inflammatory skin conditions (Jayakumar et al. 2022).

#### Photoprotection

Ultraviolet radiation, particularly UVA (320–400 nm) and UVB (280–320 nm), induces oxidative injury by generating ROS, causing direct DNA damage, and impairing the antioxidant defense system. Chronic UV exposure increases the risk of skin malignancies and accelerates photoaging (Pellacani et al. 2024).

ESC provides photoprotection through multiple mechanisms. It absorbs UV light via the benzopyranone chromophore, directly neutralizes reactive oxygen species (ROS), and upregulates antioxidant enzymes via the Nrf2/ARE pathways, thereby boosting cellular antioxidant defenses (Jayakumar et al. 2022). Xenograft studies with UV-exposed skin show that ESC decreases oxidative stress markers, such as lipid peroxidation and protein carbonyls, and helps maintain skin structure, including collagen and elastin (Kim et al. 2008).

#### Wound healing

Esculetin promotes wound healing through multiple mechanisms, including enhanced keratinocyte migration into the wound, which is critical for reepithelialization; increased expression of VEGF and TGF-β1, which support angiogenesis and extracellular matrix remodeling; and increased collagen synthesis, which aids recovery of tissue strength (Çınar et al. 2025; Garg et al. 2022). Preclinical wound-healing models show improved tissue structure, increased vascularity, and faster healing with ESC treatment compared to untreated controls (Çınar et al. 2025).

Dermatological benefits are supported by limited data, mainly 2–3 independent studies from in vitro and topical animal models. Applying these findings to human dermatological conditions needs further clinical research.

### Anti-inflammatory and antioxidant effects

Chronic inflammation and oxidative stress constitute pathogenic mechanisms in numerous diseases (cardiovascular disease, cancer, neurodegeneration, metabolic syndrome). Understanding ESC’s mechanisms for mitigating these processes is central to its therapeutic potential (Zhang et al. 2022a, b).

#### NF-κB pathway inhibition

In lipoteichoic acid (LTA)-stimulated RAW 264.7 macrophages, LTA increased nitric oxide (NO) production, elevated inducible NO synthase (iNOS) and COX-2 expression, and enhanced NF-κB p65 phosphorylation. ESC treatment in a concentration-dependent manner (2.5–20 µM) significantly reduced NO production and iNOS levels, restored IκBα levels, prevented NF-κB p65 phosphorylation, and blocked p65 nuclear translocation at concentrations ≥ 20 µM (Jayakumar et al. 2022). Additionally, ESC increased Nrf2 expression and decreased DPPH radical formation, confirming antioxidant activity.

In human nasal epithelial cells (HNEpCs) stimulated with histamine, ESC attenuated histamine-induced increases in IL-6, IL-8, and mucin (MUC5AC) production. Western blot analysis demonstrated that ESC inhibited NF-κB activation by reducing p65 phosphorylation and preventing IκBα degradation, establishing the relevance of NF-κB inhibition to the pathophysiology of allergic rhinitis (Sun et al. 2019).

#### Nrf2/ARE pathway activation

The Nrf2/ARE pathway represents the predominant cellular defense against oxidative stress. Normally, Nrf2 is sequestered in the cytoplasm by Keap1. Upon oxidative stress or electrophilic challenge, Nrf2 dissociates from Keap1, accumulates, translocates to the nucleus, and binds ARE sequences, initiating transcription of protective genes including HO-1, NQO1, and GSTs, substantially amplifying the cell’s antioxidant capacity (Jayakumar et al. 2022).

ESC effectively activates this pathway across diverse cell types (Jayakumar et al. 2022). In SH-SY5Y neurons, ESC activated Nrf2, increased glutathione (GSH) levels, and protected against oxidative stress and amyloid-beta (Aβ₁₋₄₂) oligomer-induced apoptosis. Inhibitor studies demonstrated that ERK1/2 and Akt signaling pathways contribute to ESC’s neuroprotective effects (Pruccoli et al. 2020). In renal cells (HEK293) exposed to t-BHP (tert-butyl hydroperoxide)-mediated oxidative stress, ESC promoted cell survival and reduced apoptosis by lowering pro-apoptotic factors and increasing anti-apoptotic factors at both mRNA and protein levels (Srirangan et al. 2025).

### Metabolic disorders: antidiabetic effects

Diabetes mellitus, characterized by chronic hyperglycemia from impaired insulin secretion or reduced insulin action, has reached pandemic prevalence. Type 2 diabetes mellitus (T2DM), comprising ~ 90% of diabetes cases, is driven by insulin resistance (IR) and β-cell dysfunction, with oxidative stress and chronic inflammation as key pathogenic factors (Garg et al. 2022).

#### Glucose metabolism modulation

ESC affects glucose metabolism via several pathways: boosting glycolytic enzyme activity to increase glucose usage, lowering gluconeogenic enzyme expression to reduce liver glucose production, and facilitating GLUT4 translocation to the plasma membrane in muscle and fat tissue, thereby improving glucose uptake in response to insulin (Garg et al. 2022).

#### Insulin sensitivity enhancement

Insulin resistance in T2DM involves impaired insulin receptor signaling, often caused by increased inflammation and oxidative stress. ESC enhances insulin sensitivity by inhibiting NF-κB, thereby decreasing pro-inflammatory cytokine production and reducing chronic inflammation, and by activating the PI3K/Akt pathway, which is crucial for insulin-driven glucose uptake and metabolic switching (Kim and Park 2025). Preclinical studies using diabetic animal models consistently show that ESC treatment improves glucose tolerance and insulin sensitivity, leading to lower fasting blood glucose levels and better long-term glycemic control, as indicated by HbA1c (Yang et al. 2024).

#### Pancreatic β-cell preservation

Preservation of pancreatic β-cell function is critical for maintaining insulin secretion and preventing progression to overt diabetes. ESC protects β-cells through antioxidant and anti-inflammatory mechanisms. It reduces ROS to prevent hyperglycemia-induced oxidative damage, prevents apoptosis by modulating pro- and anti-apoptotic proteins (Bcl-2/BAX ratio), and inhibits NF-κB, reducing pro-inflammatory signaling and preserving β-cell function (Garg et al. 2022).

#### Epigenetic modulation

Emerging research indicates that ESC influences histone post-translational modifications (PTHMs) involved in diabetes-related organ damage. In conditions such as type 2 diabetic nephropathy (T2DN) and insulin resistance (IR), PTHMs regulate the increased production of monocyte chemoattractant protein-1 (MCP-1) and transforming growth factor-beta (TGF-β). In experiments with Wistar rats fed a high-fat diet or treated with streptozotocin (STZ), ESC offered kidney protection by altering the renin-angiotensin system, decreasing histone H3 hyperacetylation at MCP-1 and TGF-β gene promoters, and increasing H2AK119 monoubiquitination (a repressive mark) at these sites (Kushwaha et al. 2024). While these epigenetic modifications offer a new potential mechanism, more research is needed to independently confirm these findings.

#### Mitophagy in diabetic complications

Recent investigation of mitochondrial quality control in diabetes comorbidities revealed that ESC enhanced mitophagy (selective autophagy of damaged mitochondria) in renal tubular epithelial cells exposed to hypoxia-reoxygenation injury. This involved increased expression of PINK1 and Parkin (mitophagy markers), reduced oxidative stress via activation of the Nrf2/Keap1 pathway, and preserved mitochondrial integrity (Dagar et al. 2025). While promising, this mechanism is based on a recent study and requires replication.

### Bioavailability, metabolism, safety, and formulation challenges

A critical translational barrier to ESC therapeutic development is the pronounced mismatch between preclinical efficacy (at 10–100 µM) and achievable human exposure (likely < 1 µM due to poor bioavailability). This represents the single most significant obstacle to clinical translation (Kwak et al. 2021).

#### Pharmacokinetic limitations

Esculetin has very low water solubility due to its lipophilic coumarin structure and limited hydrogen bonding with water. This significantly hampers its dissolution in the gastrointestinal tract, reducing oral bioavailability. Additionally, passive permeability through the intestinal epithelium is limited, and P-glycoprotein (MDR1) actively effluxes ESC back into the intestinal lumen, further decreasing absorption (Zhang et al. 2022a, b).

ESC is rapidly metabolized in phase II reactions, mainly through glucuronidation by UDP-glucuronosyltransferases (especially UGT1A6 and UGT1A9) and sulfation by sulfotransferases (SULTs). These conjugation processes lower lipophilicity, promote excretion, but also reduce the activity of the metabolites. In rodent studies, ESC has a short plasma half-life of 2–4 h, indicating quick hepatic metabolism and renal clearance of conjugates (Kwak et al. 2021; Wang et al. 2016).

Initial data estimate oral bioavailability in rodents at around 5–15%. However, no bioavailability studies in humans have been performed; thus, rodent data may not accurately reflect potential human absorption (Kwak et al. 2021).

#### Recent bioavailability enhancement innovations

Recognition of bioavailability limitations has motivated the development of ESC cocrystals with nicotinamide (NAM), a FDA Generally Recognized As Safe (GRAS) excipient. The ESC-NAM cocrystal improves water solubility 3–fourfold and oral bioavailability 2–threefold compared to amorphous ESC. DPPH antioxidant assays demonstrated synergistic antioxidant effects in cocrystal formulations. In rat studies, the cocrystal exhibited superior hepatoprotective effects, highlighting cocrystallization as a pragmatic bioavailability-enhancement strategy using recognized, safe excipients (F. Liu et al. 2023).

Multiple nanoparticle formulations have been explored. The chitosan nanoparticles enhance cellular uptake in macrophages while retaining anti-inflammatory activity; solid lipid nanoparticles (SLNs) improve dissolution and tissue distribution; and liposomal formulations enhance circulation time and tissue delivery (Garg et al. 2022). Most nanoparticle systems remain at the preclinical stage; in vivo bioavailability data are limited, and regulatory pathways for nanotechnology-based nutraceuticals remain undefined.

Rational structural optimization at C-4, C-7, and C-8 positions enhances metabolic stability. The 8-(pyrrolidin-1-ylmethyl)−4-trifluoromethyl ESC derivative exhibited a threefold longer half-life in human liver S9 fractions than the parent ESC, demonstrating the feasibility of improving pharmacokinetics through design (Choi et al. 2023).

#### Safety and toxicological assessment

Intraperitoneal LD₅₀ in rodents ranges from 300 to 400 mg/kg, indicating moderate acute lethality (e.g., i.p. LD₅₀ ~ 1450 mg/kg in rats; oral LD₅₀ < 2000 mg/kg). No published oral acute toxicity studies in animals or humans are available (Anywar and Muhumuza 2023). Limited 28-day oral gavage studies in rats at doses up to 100 mg/kg/day show no obvious adverse clinical signs, body weight changes, or gross organ pathology upon autopsy (Pullaiah et al. 2021). However, no detailed histopathological examination, clinical chemistry, or hematology data have been published. No chronic toxicity studies (≥ 90 days) have been published in any animal species. This represents a critical gap for any compound intended for chronic human use.

Special safety considerations include the pro-oxidant potential at high concentrations, which conflicts with antioxidant claims; possible weak estrogenic activity in hormone-sensitive groups; CYP450 inhibition at higher levels, which raises the risk of drug interactions; and the inherent phototoxicity of coumarin scaffolds (Garg et al. 2022).

In pharmaceutical development, an IND-enabling toxicology package typically includes GLP-compliant 28-day and 90-day oral toxicity studies, genotoxicity testing, and reproductive toxicity assessments to identify a NOAEL (Antunović et al. 2011). However, such a package has not been developed for esculetin. For nutraceutical development, the New Dietary Ingredient Notification (NDIN) process requires reasonable assurance of safety at intended use levels (Komala et al. 2023). Presently, existing data do not meet the requirements for either regulatory pathway.

### Cardiovascular effects

**Fig. 2:**
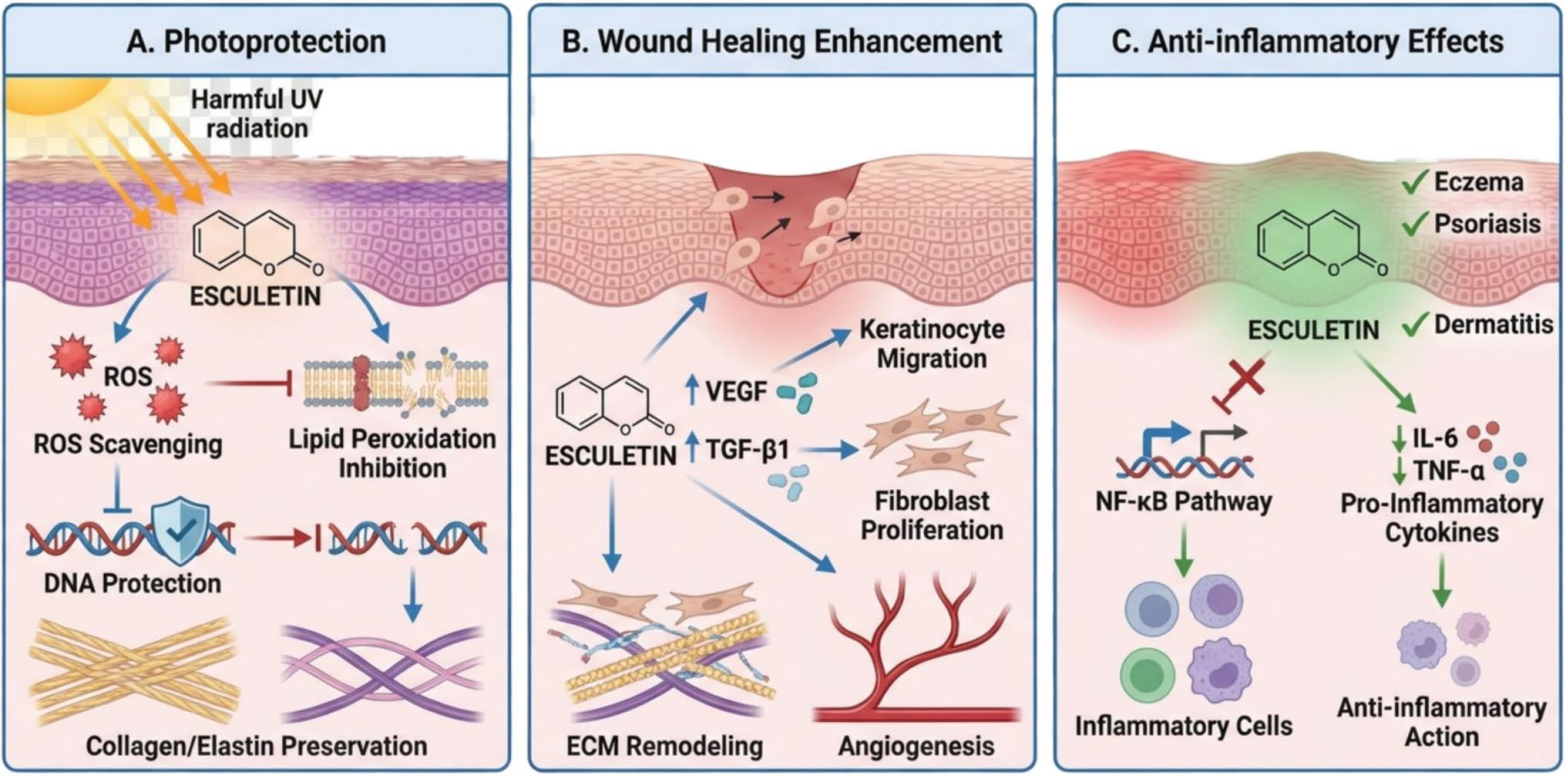
Esculetin’s multitarget cardiovascular protective mechanisms

Cardiovascular disease (CVD), encompassing coronary artery disease, hypertension, heart failure, arrhythmias, and stroke, remains the leading cause of morbidity and mortality globally. Risk factors (hypertension, dyslipidemia, smoking, hyperglycemia, obesity) promote chronic inflammation and oxidative stress, driving atherosclerosis and myocardial damage (Wang et al. 2023) (Fig. 2).

#### Cardioprotective effects

Oral pretreatment with ESC exhibits anti-lipoperoxidative and antioxidant effects in isoproterenol-induced myocardial infarction (MI) models in rats, preventing MI through free radical scavenging (Jabbar Mujbel et al. 2025; Mujbel et al. 2025). Research confirms that ESC provides multitarget cardiovascular protection- anti-atherosclerotic, anti-ischemic, antiplatelet, and endothelial-protective effects through coordinated modulation of Nrf2, NF-κB, MAPKs, JAK2/STAT3, SIRT3/AMPK/mTOR, PLC/PKC/Akt, PI3K/Akt, cyclic nucleotides, and NLRP3 pathways. Human clinical confirmation is still lacking (Hsia et al. 2019; Lee 2021; Wang et al. 2022).

#### Effects on lipid metabolism

In vitro studies indicate that ESC, at concentrations ranging from 12.5 to 100 µM, decreases lipid accumulation in 3T3-L1 adipocytes in a dose-dependent manner. This effect results from downregulation of key differentiation markers (PPARγ, C/EBPα) and enhanced phosphorylation of AMP-activated protein kinase (AMPK) and acetyl-CoA carboxylase (ACC), with complementary antioxidant effects contributing to its anti-adipogenic profile, which is critical for lipid metabolism control (Kim and Lee 2015; Xia et al. 2023).

#### Anti-atherosclerotic properties

Atherosclerosis development is strongly associated with oxidized low-density lipoprotein oxLDL), which promotes foam cell formation and vascular inflammation. In vitro, ESC at concentrations of 0.1–1 mM inhibited LDL oxidation mediated by copper ions and nitric oxide donors in a dose-dependent manner (Lee et al. 2002). Previous research demonstrates that ESC alleviates hyperlipidemia by decreasing lipid accumulation, restoring serum HDL-C levels, and inhibiting MMP-9 production and LDL oxidation (Wang et al. 2022). In mice fed a high-fat diet, esculetin raises postprandial HDL cholesterol levels to levels similar to those of statins and promotes cholesterol excretion via bile. It also boosts CD36-mediated uptake of oxLDL by adipose tissue macrophages and promotes HDL production (Wang et al. 2025).

#### Antithrombotic properties

Recent investigations show that ESC and esculin pentasulfate (EPS), a heavily sulfated esculetin derivative, significantly reduced thrombus weight and length, prolonged APTT, PT, and TT, and confirmed strong systemic anticoagulant activity in rat venous thrombosis models induced by inferior vena cava ligation. They also extended the activated clotting time and slowed the clotting rate compared to controls. ESC pentasulfate demonstrated superior anticoagulant effects relative to parent ESC, with additional antiplatelet activity (Ahmad et al. 2018). These findings suggest that structural modification enhances antithrombotic effectiveness.

In washed human platelets, esculetin at concentrations of 10–80 µM inhibits collagen and arachidonic acid–induced aggregation, ATP release, P-selectin expression, hydroxyl radical production, and PLCβ2–PKC–Akt signaling (Hsia et al. 2019). Additional research indicates that esculetin inhibits U46619- or collagen-induced platelet activation by increasing cAMP/cGMP levels, reducing calcium mobilization, impairing αIIbβ3-fibrinogen binding, and decreasing clot retraction (Park and Lee 2021).

Esculetin (ESC) provides comprehensive cardiovascular protection by coordinating the modulation of multiple pathways. Central heart illustration shows protection against myocardial infarction and ischemia via antioxidant mechanisms. Four mechanistic panels depict(1) atherosclerosis prevention—inhibition of oxLDL and foam cell formation, HDL-C restoration, cholesterol excretion, and MMP-9 suppression; (2) molecular signaling—Nrf2 activation, NF-κB suppression, and modulation of MAPK, JAK2/STAT3, SIRT3/AMPK/mTOR, PI3K/Akt, and NLRP3 pathways reducing oxidative stress and inflammation; (3) lipid metabolism—AMPK/ACC phosphorylation and PPARγ/C/EBPα downregulation reducing adipocyte lipid accumulation; (4) antithrombotic activity—platelet aggregation inhibition, anticoagulation (prolonged APTT/PT/TT), cAMP/cGMP elevation, and PLCβ2-PKC-Akt pathway blockade. Green arrows indicate activation; red arrows/symbols indicate inhibition. Evidence from preclinical in vitro and in vivo models.

### Neurological and neurodegenerative conditions

**Fig. 3:**
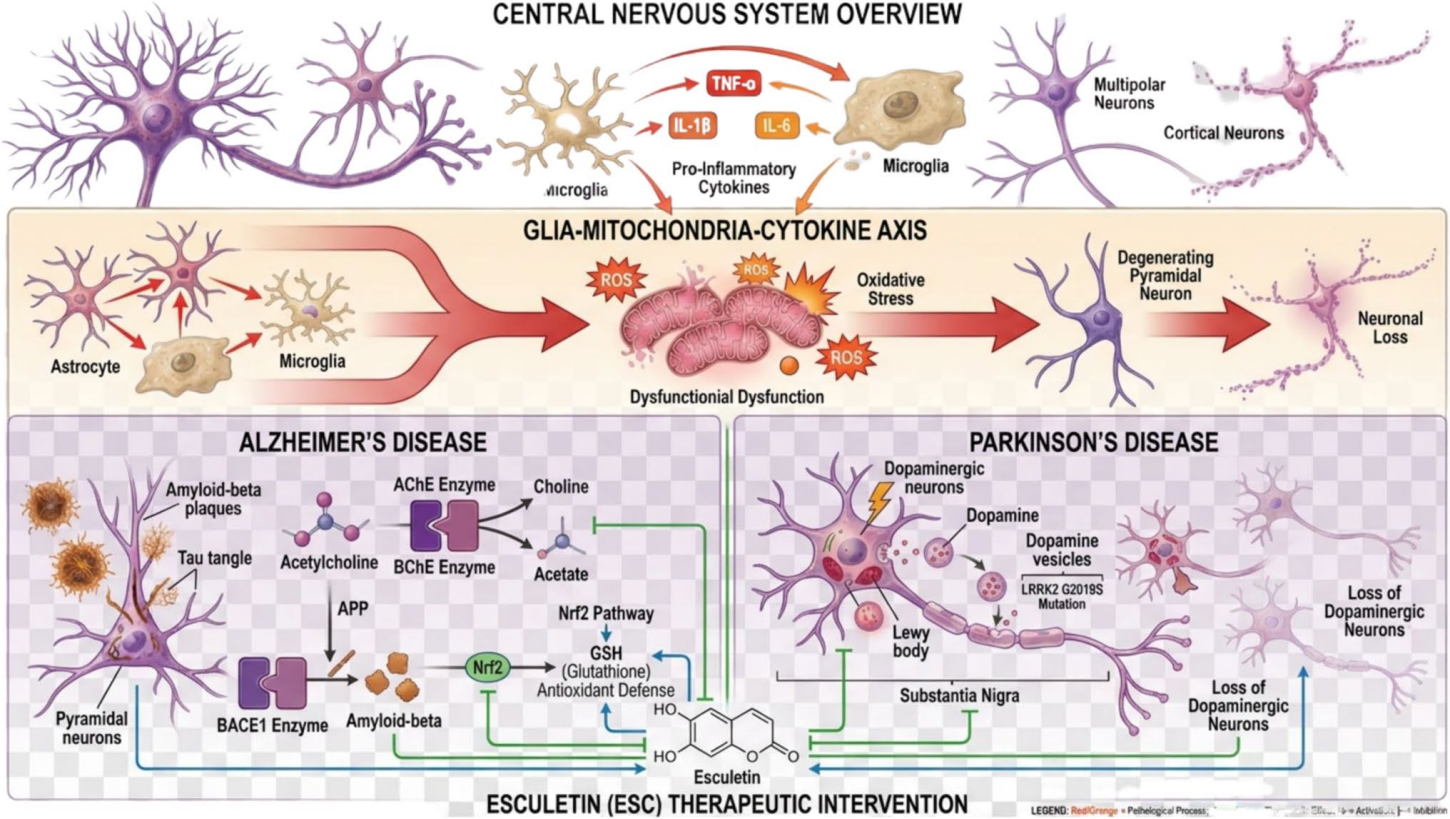
Multi-target therapeutic mechanisms of esculetin in Alzheimer’s and Parkinson’s diseases

Central nervous system (CNS) diseases, including Alzheimer’s disease (AD), Parkinson’s disease (PD), cerebral ischemia, and traumatic brain injury, are characterized by chronic neuroinflammation, oxidative stress, and mitochondrial dysfunction. Astrocytes and activated microglia release pro-inflammatory cytokines (TNF-α, IL-1β, IL-6), contributing to neuronal loss. This self-reinforcing glia–mitochondria–cytokine axis is now a major therapeutic target for modifying disease progression (Qin et al. 2024). These mechanisms are schematically illustrated in Fig. 3 and discussed in detail below.

#### Alzheimer’s disease

Oxidative stress significantly worsens AD pathology by weakening antioxidant defenses (e.g., glutathione and Nrf2). ESC functions as a dual-action antioxidant, reducing ROS directly and promoting antioxidant gene expression through Nrf2 activation. In SH-SY5Y neurons, ESC activated Nrf2, increased GSH levels, and protected against oxidative stress and Aβ₁₋₄₂ oligomer-induced apoptosis (Pruccoli et al. 2020).

Regarding enzyme inhibition, the cholinergic hypothesis suggests that excessive proteolytic activity may contribute to the development of AD (V Schulz 2003). Current evidence supports esculetin as a promising multi-target enzyme inhibitor against acetylcholinesterase (AChE), which breaks down acetylcholine (IC₅₀ = 6.13 µM), as well as on butyrylcholinesterase (BChE), involved in neurotransmitter breakdown (IC₅₀ = 8.66 µM), and on β-site amyloid precursor protein cleaving enzyme (BACE1), critical for producing amyloid-beta (IC₅₀ = 7.67 µM) (Ali et al. 2016). However, these results are limited to in vitro studies; further, in vivo research in animals or humans is necessary to confirm efficacy, selectivity, brain exposure, and safety.

In STZ-induced AD rats, ESC at doses of 25 and 50 mg/kg showed notable neuroprotection by activating Nrf2, increasing GSH levels, and stimulating ERK and Akt pathways while also preventing Aβ buildup (Garg et al. 2022; Pruccoli et al. 2020). These actions helped reduce STZ-induced neurotoxicity in the hippocampus, which is essential for memory function.

#### Parkinson’s disease

The G2019S mutation in leucine-rich repeat kinase-2 (LRRK2) is the leading cause of inherited PD. In vitro studies demonstrated that ESC, at concentrations of 0.001–10 µM, exhibited strong kinase-inhibitory effects against LRRK2, with effects that increased in a dose-dependent manner (Angeles et al. 2016). Additionally, ESC decreased dopaminergic neuron loss, enhanced antioxidant activity, and reduced locomotor performance associated with the G2019S mutation (Skalicka-Woźniak et al. 2016). However, unlike optimized clinical LRRK2 inhibitors, esculetin’s pharmacokinetics, brain exposure, and safety in humans with PD remain unknown, so its role is currently limited to proof-of-concept rather than a validated therapeutic.

Schematic overview of neurodegenerative pathophysiology and esculetin intervention.Top: activated microglia and astrocytes release pro-inflammatory cytokines (TNF-α, IL-1β, IL-6) in the central nervous system.Middle: the glia-mitochondria-cytokine axis drives neurodegeneration through ROS-mediated oxidative stress and mitochondrial dysfunction (red arrows indicate pathological progression).Bottom left (Alzheimer’s disease): amyloid-beta plaques and tau tangles characterize AD pathology. BACE1 cleaves APP to generate Aβ; AChE and BChE degrade acetylcholine. Nrf2 activation promotes GSH-mediated antioxidant defense.Bottom right (Parkinson’s disease): dopaminergic neurons in the substantia nigra accumulate Lewy bodies and undergo degeneration. LRRK2 G2019S mutation increases pathological kinase activity.Bottom (esculetin intervention):ESC exhibits multi-target effects (green/blue arrows): enzyme inhibition (AChE IC₅₀ = 6.13 µM; BChE IC₅₀ = 8.66 µM; BACE1 IC₅₀ = 7.67 µM; LRRK2 0.001–10 µM) and Nrf2 pathway activation. Red/orange indicates pathological processes; green/blue indicates therapeutic effects. AChE, acetylcholinesterase; APP, amyloid precursor protein; Aβ, amyloid-beta; BACE1, β-site APP cleaving enzyme 1; BChE, butyrylcholinesterase; ESC, esculetin; GSH, glutathione; LRRK2, leucine-rich repeat kinase 2; Nrf2, nuclear factor erythroid 2-related factor 2; ROS, reactive oxygen species.

### Hepatoprotection

The liver plays a crucial role in drug metabolism and detoxification. Several hepatotoxicity models have been used to assess ESC’s protective effects.

#### Acetaminophen-induced hepatotoxicity

In acetaminophen (APAP)-induced hepatotoxicity models, ESC pre-treatment reduced liver injury markers (ALT, AST) and improved hepatocyte survival. ESC prevented hepatocyte apoptosis by reducing the Bcl-2-to-BAX ratio and inhibiting caspase-3 activation. Additionally, ESC enhanced hepatic antioxidant enzyme expression (SOD, CAT, GPx), supporting phase I and II detoxification capacity (Lin et al. 2000).

#### ESC-nicotinamide cocrystal

The ESC-nicotinamide cocrystal exhibited superior in vitro and in vivo hepatoprotective performance relative to amorphous ESC. The cocrystal demonstrated enhanced protective effects in rat models, suggesting that bioavailability enhancement translates into improved hepatoprotective efficacy in vivo (Kim et al. 2025; Liu et al. 2023).

#### Anti-HBV derivatives

Recent studies demonstrated anti-HBV activity of ESC derivatives in HepG2.2.15 cells, with specific derivatives reducing HBeAg and HBsAg expression by day 9 and stronger inhibition than lamivudine (3TC) under the same dosing schedule, with lower cytotoxicity (Huang et al. 2019; Ye et al. 2023). However, the efficacy of derivatives compared with the standard antiviral lamivudine remains unexplored.

Hepatoprotection is moderately supported by multiple studies in acute toxicity models; however, all evidence comes from acute hepatotoxicity models in rodents, not chronic liver disease (Ye et al. 2023). Data on anti-HBV derivatives remains preliminary. More head-to-head animal studies and mechanism research are needed before their therapeutic value compared to nucleostide analogues can be determined.

### Renal protection

#### General renal antioxidant effects

In renal epithelial cells (HEK293) exposed to tert-butyl hydroperoxide (tBHP)-mediated oxidative stress, ESC demonstrated antioxidant activity by promoting cell survival and reducing apoptosis. ESC lowered pro-apoptotic factors (BAX, caspase-3) and increased anti-apoptotic factors (Bcl-2, Bcl-xL) at both mRNA and protein levels (Jung et al. 2022a, b). Similar anti-apoptotic and antioxidant profiles, characterized by lower Bax and caspase-3 levels and increased Bcl-2 levels, are observed in ESC-treated retinal pigment epithelial cells under t-BHP stress, indicating a shared mechanism across epithelial tissues (Jung et al. 2022b).

In lupus nephritis–prone MRL/lpr mice, ESC alleviated renal dysfunction and structural damage by lowering blood urea nitrogen, serum creatinine, albuminuria, glomerular hypertrophy, fibrosis, and inflammatory infiltration. Mechanistically, ESC activated Nrf2-dependent antioxidant pathways and decreased renal oxidative stress. Moreover, it inhibited complement activation at the level of the C3 convertase (C4b2a) and suppressed NF-κB and TGF-β/Smad3 profibrotic signaling (Y. Zhang et al. 2022a, b).

Similarly, the ESC glycoside esculin helped prevent renal ischemia–reperfusion injury by decreasing oxidative stress, Bax, and caspase-3 levels while restoring Bcl-2. This further supports mitochondria-focused anti-apoptotic protection of the kidneys (El-Maadawy et al. 2022).

#### Diabetic nephropathy and epigenetic modulation

In high-fat diet and low-dose STZ Wistar rats (type 2-like model), ESC provided kidney protection by modulating the renin-angiotensin system, decreasing histone H3 hyperacetylation at the MCP-1 and TGF-β promoters (H3K14ac, H3K18ac), and increasing H2AK119 monoubiquitination at these gene sites. These changes coincided with decreased MCP-1/TGF-β1 expression and reduced overactivity of the renin–angiotensin system (RAS), indicating that ESC connects RAS regulation to targeted epigenetic silencing of inflammatory and fibrotic pathways programs (Kadakol et al. 2017). Previous studies in STZ-type 1 diabetic rats revealed that ESC also reverses the global loss of H3K9/14 acetylation and H3K4 monomethylation, including at Mmp13/Bmp6 sites, leading to normalization of profibrotic signaling and glomerulosclerosis (Surse et al. 2011). These epigenetic modifications suggest a new mechanism that needs further verification.

HDAC inhibitors, BET inhibitors, and SIRT1 modulators are under investigation as therapies for diabetic nephropathy (Kushwaha et al. 2022; Lu et al. 2021). However, most of these agents act broadly and pose toxicity risks. ESC seems to induce more selective changes at the promoter level in the kidney and heart within diabetic models, but this has not yet been evaluated in humans (Kadakol et al. 2017).

#### Acute kidney injury in diabetes

In T1DM mice with ischemia–reperfusion-induced acute kidney injury (AKI), ESC (50, 100 mg/kg), ESC enhanced mitophagy in renal tubular epithelial cells (NRK-52E) subjected to hypoxia-reoxygenation injury, characterized by increased levels of PINK1 and Parkin (mitophagy markers), reduced oxidative stress via the Nrf2/Keap1 pathway, cell apoptosis, and maintained mitochondrial integrity (Dagar et al. 2024). Combining esculetin with phloretin in diabetic AKI rats further enhanced mitophagy by increasing PINK1, Parkin, LC3B, and decreasing p62. It also reduced inflammation, lowering TLR4/NF‑κB, TNF‑α, and MCP‑1 levels, providing better protection than either agent alone (Dagar et al. 2025).

### Anticancer effects

**Fig. 4:**
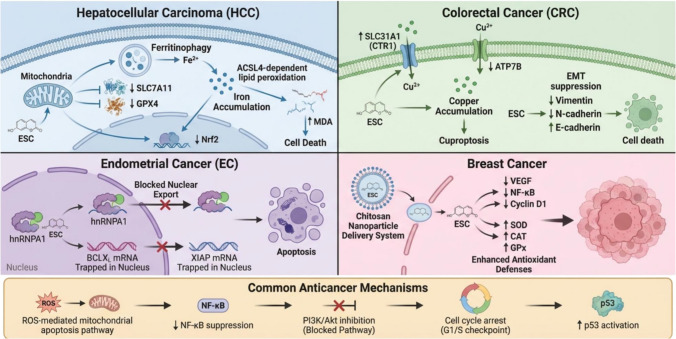
Anticancer effects of esculetin across multiple cancer types

Cancer is marked by abnormal cell growth, resistance to cell death, and the ability to invade and spread. Several signaling pathways contribute to malignant transformation, including oncogenic pathways (RAS, PI3K/Akt, Wnt/β-catenin), anti-apoptotic Bcl-2 family activity, and epithelial-mesenchymal transition (EMT)/EMP-driven invasion and resistance (Kaloni et al. 2023; Nie et al. 2025; Sanchez-Vega et al. 2018). These mechanisms are schematically illustrated in Fig. 4 and discussed in detail below.

#### Common anticancer mechanisms

Across various cancer types, esculetin consistently triggers ROS-mediated mitochondrial apoptosis, suppresses NF-κB and PI3K/Akt survival pathways, induces cell cycle arrest, and frequently involves p53-axis regulation, reinforcing its role as a multitarget anticancer agent in preclinical studies (Garg et al. 2022).

#### Hepatocellular carcinoma: ferroptosis mechanism

Recent evidence shows that ESC induces ferroptosis, an iron-dependent form of cell death distinct from apoptosis, in HUH7 and HCCLM3 hepatocellular carcinoma cells. Ferroptosis occurs when antioxidant defenses are insufficient, leading to excessive lipid peroxidation. ESC triggers ferroptosis by promoting ferritinophagy, which increases autophagy-mediated ferritin breakdown and releases iron; enhances lipid peroxidation through ACSL4-dependent pathways; and decreases antioxidant protection by reducing ferritin and SLC7A11 levels (Tang et al. 2021; Xiu et al. 2023; Yan et al. 2021).

Similar mechanisms—such as SLC7A11/GPX4 inhibition, ferritinophagy, and Nrf2 suppression—are established ferroptosis pathways in HCC, supporting the biological plausibility of the ESC findings (An et al. 2025; Jiang et al. 2024). A 2025 study independently confirms that ESC can also induce ferroptosis by inhibiting the Nrf2–xCT/GPX4 axis in HCC cells, leading to Fe accumulation, higher MDA levels, reduced GPX4, and reversal with ferrostatin-1 (Qu et al. 2025).

In vivo xenograft models demonstrated that ESC decreased tumor growth, increased lipid peroxidation (MDA levels), elevated ferritinophagy markers (LC3-II, NCOA4), and reduced antioxidant protein expression. This ferroptosis mechanism represents a notable discovery, as ferroptosis is an understudied modality in cancer therapeutics and may enable targeting of apoptosis-resistant cancers (Xiu et al. 2023). However, this finding derives from one detailed 2023 ferritinophagy paper and a 2025 Nrf2–xCT/GPX4 study in HCC models (Qu et al. 2025; Xiu et al. 2023). Independent replication, off-target profiling, and assessment in sorafenib-resistant and normal hepatocytes are needed before translational conclusions.

#### Colorectal cancer: copper homeostasis disruption

Recent work suggests ESC may exploit copper dysregulation in colorectal cancer (CRC), complementing its known pro-apoptotic and anti-EMT actions. ESC selectively inhibits CRC cell growth, with lower IC₅₀ values in cancer cells than in normal colonic epithelial cells. At the molecular level, ESC increases SLC31A1 (CTR1) expression, a copper importer, thereby boosting copper uptake in cancer cells. It also decreases ATP7B expression, a copper exporter, hindering copper efflux (Guo et al. 2024). These combined effects lead to copper accumulation to toxic levels, triggering cuproptosis (Liu and Tang 2022; Wang et al. 2024; Xie et al. 2023). However, the Guo study shows “copper-induced cell death” linked to transporter changes; since hallmark cuproptosis features such as DLAT aggregation and Fe–S protein loss were not observed, the classification as cuproptosis remains tentative rather than conclusively proven.

Additionally, multiple studies have shown that esculetin/esculin inhibits colony formation, promotes apoptosis, and induces cell cycle arrest in S phase or G0/G1 by decreasing Vimentin and N-cadherin (markers of EMT) while increasing E-cadherin (an epithelial marker), and strongly inhibits in vivo tumor growth in CRC (Ji et al. 2024; Ma et al. 2025; Sabouni et al. 2023). This action suppresses epithelial-mesenchymal transition. In vivo xenograft experiments showed that ESC reduced tumor size and lowered Ki-67 (a proliferation marker). This copper-homeostasis mechanism is entirely new in the ESC literature, offering a uniquely selective strategy against CRC (Guo et al. 2024). However, this evidence is based on a single 2024 study, and independent verification is necessary.

#### Endometrial cancer: hnRNPA1-mRNA export blocking

A recent study highlights a unique mechanistic approach to ESC in endometrial cancer, involving nuclear export inhibition. ESC suppresses cell growth and triggers apoptosis in EC cell lines. Proteomic analysis using LC–MS/MS identified heterogeneous nuclear ribonucleoprotein A1 (hnRNPA1) as a direct target of ESC (Ruqi Jiang et al. 2021a, b). Normally, hnRNPA1 assists in the export of mRNA encoding anti-apoptotic proteins such as BCLXL and XIAP. ESC binds to hnRNPA1, blocking the export of hnRNPA1/mRNA complexes from the nucleus and thereby preventing the expression of BCLXL and XIAP at both the transcriptional and translational levels. This results in apoptosis induction and reduced cell proliferation (Jin et al. 2024; Lu et al. 2022). In xenograft models, ESC reduced tumor growth and decreased BCL-XL and XIAP levels (Ruqi Jiang et al. 2021a, b).

In pancreatic and ovarian cancers, ESC primarily influences the KEAP1–Nrf2/ROS pathways or transcriptional networks to promote apoptosis and cell cycle arrest, without reported targeting of hnRNPA1. The EC study, therefore, presents a different mechanistic approach: rather than modifying signaling pathways or redox states, ESC prevents the nuclear export of survival mRNAs (Arora et al. 2016; Fan et al. 2025).

#### Breast cancer

In a DMBA-induced mammary carcinoma rat model, oral esculetin-loaded chitosan nanoparticles (ESC-CNPs) (25–100 mg/kg) markedly inhibited tumor development. The treatment reduced tumor incidence, volume, and number of nodules compared with DMBA alone. Moreover, it maintained biochemical stability and lowered levels of pro-tumor factors, including VEGF, NF-κB, and Cyclin D1. Additionally, ESC-CNPs enhanced antioxidant defenses, raising SOD, CAT, and GPx levels and improved lipid profiles while decreasing lipid peroxidation. Histopathological and molecular evidence support significant chemopreventive effects against breast cancer (Thirugnanam et al. 2025).

A supplementary study using the same DMBA model demonstrated that ESC-CNPs also protected liver from oxidative and enzymatic damage related to carcinogenesis, reinforcing their systemic antioxidant and detoxification roles (Thirugnanam and Ragunathan 2024). Chitosan-based nanoparticles are well-known for improving the oral bioavailability and tumor targeting of poorly soluble natural antioxidants in breast cancer models (Herdiana et al. 2023).

The anticancer effects are strongly aligned with well-supported antioxidant and NF-κB–modulating mechanisms seen across esculetin research. Newly proposed mechanisms (ferroptosis, cuproptosis, and hnRNPA1 export inhibition) are not yet demonstrated in breast cancer and remain speculative until replicated and extended beyond single-cell or animal studies.

Esculetin (ESC) exhibits diverse anticancer mechanisms across different malignancies.(Top left)In hepatocellular carcinoma (HCC), ESC induces ferroptosis through multiple pathways: promoting ferritinophagy-mediated iron release from ferritin, suppressing antioxidant defenses (↓SLC7A11, ↓GPX4, ↓Nrf2), enhancing ACSL4-dependent lipid peroxidation, and increasing malondialdehyde (MDA) levels, ultimately leading to iron-dependent cell death.(Top right)In colorectal cancer (CRC), ESC disrupts copper homeostasis by upregulating the copper importer SLC31A1 (CTR1) and downregulating the copper exporter ATP7B, resulting in toxic copper accumulation and cuproptosis. Additionally, ESC suppresses epithelial-mesenchymal transition (EMT) by decreasing mesenchymal markers (Vimentin, N-cadherin) and increasing epithelial marker (E-cadherin).(Bottom left)In endometrial cancer (EC), ESC directly binds to heterogeneous nuclear ribonucleoprotein A1 (hnRNPA1), blocking nuclear export of anti-apoptotic mRNAs (BCLXL, XIAP), which remain trapped in the nucleus, thereby preventing their translation and inducing apoptosis.(Bottom right)In breast cancer, ESC delivered via chitosan nanoparticles reduces pro-tumorigenic factors (VEGF, NF-κB, Cyclin D1) while enhancing antioxidant defenses (SOD, CAT, GPx), leading to tumor growth inhibition.(Bottom panel)Common anticancer mechanisms shared across cancer types include ROS-mediated mitochondrial apoptosis, NF-κB suppression, PI3K/Akt pathway inhibition, cell cycle arrest at G1/S checkpoint, and p53 activation. Arrows indicate pathway direction; ↑ indicates upregulation; ↓ indicates downregulation; red X indicates blockade or inhibition.

### Antimicrobial, antifungal, and antiviral properties

#### Antibacterial activity

ESC exhibits potent anti‑virulence activity against multidrug-resistant (MDR) strains, mainly by inhibiting the type III secretion system (T3SS1) in *Salmonella* and quorum sensing/biofilms in *Aeromonas* while also protecting the intestinal barrier. In infected animals, ESC reduced inflammation, restored goblet cell and tight junction proteins, helped reestablish microbial balance, and decreased cecal tissue damage by activating Nrf2 signaling (Xu et al. 2024).

Against *Aeromonas hydrophila* SHAe 115, ESC effectively blocks quorum sensing (QS) and prevents biofilm formation. At concentrations ranging from 25 to 100 µg/mL, it reduces protease and hemolysin production, disrupts swarming motility, and disrupts biofilm biomass and architecture (confirmed by CLSM/SEM). qRT-PCR analysis showed decreased expression of quorum-sensing genes and increased litR expression, both of which inhibit biofilm development (Sun et al. 2021).

#### Antiviral activity

Esculetin shows promising antiviral effects, especially against the hepatitis B virus (HBV) and, via derivatives/related systems, against the infectious bronchitis virus (IBV). ESC prevents the replication of IBV in tracheal, pulmonary, and intestinal tissues by upregulating type I interferons (IFN‑α/β) via MAVS/MDA5 and dampening NF‑κB-mediated inflammation in trachea and lung, indicating its therapeutic potential (He et al. 2023). In the hepatitis B virus, ESC derivatives reduce HBsAg, HBeAg, HBV DNA, and HBx protein in a time- and dose-dependent manner by altering the C-7 position. Specifically, morpholine groups markedly decrease HBeAg expression, while 2-methylimidazole derivatives effectively suppress HBsAg levels (SiXin et al. 2019).

### Antifungal activity

ESC is a potent antifungal that targets dermatophytes, causing fungal cell death across 84 clinical isolates of *Trichophyton* and *Microsporum* species, with MIC₁₀₀ values from 11.13 to 416.34 µg/mL. It effectively treats superficial mycoses, such as athlete’s foot, tinea corporis (ringworm), tinea cruris, and onychomycosis. Non-dermatophyte molds such as *Fusarium*, *Scopulariopsis brevicaulis*,* Aspergillus fumigatus*, and yeasts like *Candida albicans*, *C. krusei*, and* Malassezia furfur* are not inhibited at similar concentrations, demonstrating selectivity for dermatophytes. Although MICs are higher than those of standard azoles or terbinafine in vitro, the use of topical formulations at percent-level concentrations makes these MICs clinically relevant. Esculin, a glycosylated, water-soluble prodrug, becomes active only after hydrolysis to esculetin by β-glucosidases produced by dermatophytes and skin microbiota, enabling in situ activation on infected skin. Additionally, normal skin bacteria grow on esculin without inhibition, dependent on β-glucosidases, indicating minimal impact on healthy skin flora when using an esculin–esculetin system (Mercer et al. 2013).

## Limitations of current evidence and knowledge gaps

Esculetin, a natural coumarin derivative, has shown various pharmacological activities in preclinical studies, including antioxidant, anti-inflammatory, anticancer, antidiabetic, neuroprotective, and cardioprotective effects. However, moving esculetin from laboratory research to clinical use faces significant challenges due to limited current evidence. Most mechanistic studies rely on monolayer cell cultures or rodent models with esculetin concentrations (10–100 µM) much higher than those achieved in human plasma (< 1 µM). These models lack tissue architecture, vascularization, and immune microenvironments and do not simulate metabolic processes or drug interactions. Additionally, rodent CYP450 enzyme profiles and glucuronidation rates differ considerably from those of humans, leading to species-specific pharmacokinetic differences. There is limited data on human absorption and metabolism; oral bioavailability is low because of extensive glucuronidation. No clinical trials have defined safe or effective dosing. There is also considerable variability across studies in cell lines, concentrations, treatment durations, measured outcomes, animal strains, and disease models, making comparisons and meta-analyses difficult. Study design weaknesses, including small sample sizes, insufficient power calculations, and lack of preregistration, raise concerns about reproducibility and the potential for publication bias favoring positive results. In addition, most research is limited to short-term or subchronic periods (days to weeks), which are insufficient for assessing tolerance or delayed adverse effects. Xenograft models in immunocompromised hosts do not accurately replicate the human immune system. Furthermore, the absence of long-term safety data precludes any conclusions about chronic use risks.

## Conclusions and future research directions

Esculetin has multi-target pharmacology, modulating NF-κB, Nrf2/ARE, MAPK, and mitochondrial apoptosis, with reported benefits in models of cancer, diabetes, arthritis, liver, cardiovascular, and neurodegenerative disease. Disease-specific mechanisms, such as ferroptosis in HCC and apoptosis/ferroptosis in colorectal cancer, support its role as a redox-sensitive cell death modulator. Nonetheless, efficacy is shown at 10–100 µM in vitro and at relatively high doses in animals, while rat oral bioavailability is low (~ 19% in one study; generally 5–20%) and is governed by poor solubility and extensive glucuronidation. There are no clinical trials or human safety studies, and chronic toxicity, prooxidant, estrogenic, CYP-inhibitory, and phototoxic risks are essentially unknown.

## Data Availability

No datasets were generated or analyzed during this study.
